# An Effective Approach to Improving Day-Case Rates following Laparoscopic Cholecystectomy

**DOI:** 10.1155/2011/564587

**Published:** 2011-04-07

**Authors:** M. G. Clarke, T. Wheatley, M. Hill, G. Werrett, G. Sanders

**Affiliations:** ^1^Oesophagogastric Unit, Department of Upper Gastrointestinal Surgery, Derriford Hospital, Plymouth PL6 8DH, UK; ^2^Department of Anaesthesia, Derriford Hospital, Plymouth PL6 8DH, UK

## Abstract

*Background*. Day-case laparoscopic cholecystectomy (LC) is a safe and cost-effective treatment for gallstones. In 2006, our institution recorded an 86% laparoscopic, 10% day-case, and 5% readmission rate. A gallbladder pathway was therefore introduced in 2007 with the aim of increasing daycase rates. *Methods*. Patients with symptomatic gallstones, proven on ultrasound, were referred to a specialist-led clinic. Those suitable for surgery were consented, preassessed, and provided with a choice of dates. All defaulted to day case unless deemed unsuitable due to comorbidity or social factors. *Results*. The number of cholecystectomies increased from 464 in 2006 to 578 in 2008. Day-case rates in 2006, 2007, 2008, and June 2009 were 10%, 20%, 30%, and 61%, respectively. Laparoscopic and readmission rates remained unchanged. Conversion rates for elective cholecystectomy fell from 6% in 2006 to 3% in 2009. *Conclusions*. Development of a gallbladder pathway increased day-case rates sixfold without an associated increase in conversion or readmission rates.

## 1. Introduction

In 2006, the NHS Institute for Innovation and Improvement, as part of the high volume Healthcare Resource Groups (HRG) program, produced a document entitled “Focus on Cholecystectomy” which aimed to improve both the quality and value of care for patients undergoing cholecystectomy [1]. This arose following an initial audit of 49,077 cholecystectomies, performed in England between April 2005 and 2006, in which a laparoscopic rate of 50 to 90 per cent and an average day-case rate of only 6.4 per cent was identified. This outlined strategies to improve the patient pathway including aspects relating to outpatient referral, pre-, peri- and postoperative care.

**Figure 1:**
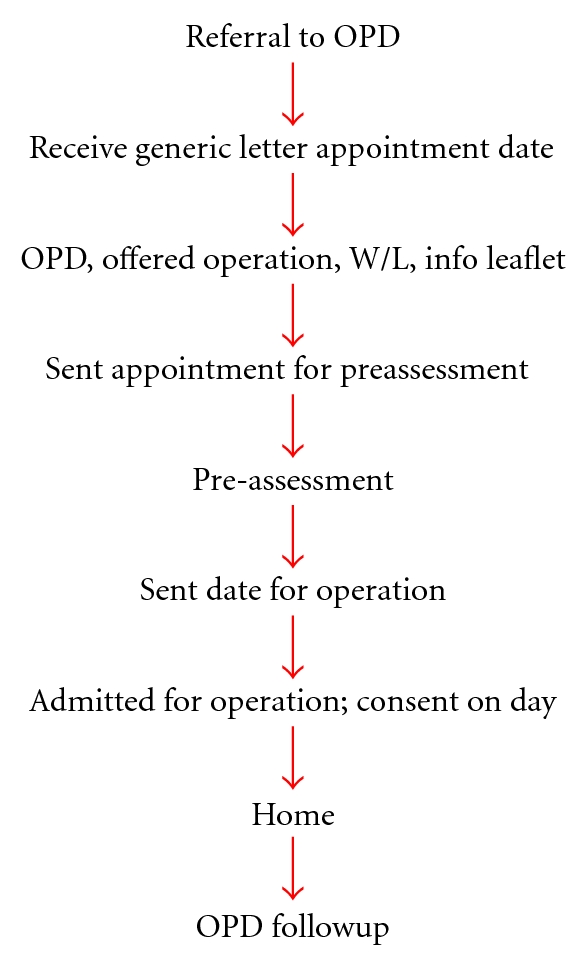
Pre-existing gallbladder patient pathway prior to 2006.

At the time of this publication, our own institution had a laparoscopic cholecystectomy rate of 86 per cent, with a day-case rate of 10 per cent and readmission rate of 5 per cent. The patient pathway (Figure 1) consisted of four patient visits, including initial outpatient appointment, preassessment clinic, day of surgery, and follow-up appointment. This study aimed to examine the impact of introducing a new gallbladder pathway, based on the “Focus on Cholecystectomy” document, on the laparoscopic rate, conversion rate, day-case rate and readmission rate following cholecystectomy.

## 2. Materials and Methods

**Figure 2:**
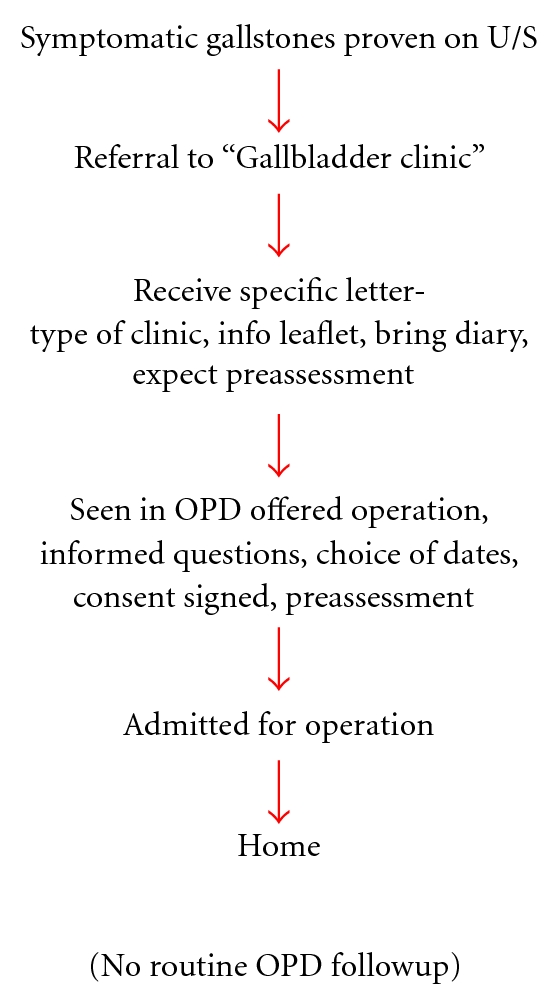
New gallbladder patient pathway introduced in February 2007.

In February 2007, a new cholecystectomy patient pathway was introduced at our institution (Figure 2). This included six stages and required only two patient visits. All 13 surgeons performing laparoscopic cholecystectomy were invited to participate. Patients with symptomatic gallstones, proven on ultrasound (USS), could be referred by their General Practitioner (GP) to a specialist-led “Gallbladder Clinic” via the choose and book system. Patients with a history of gallstone pancreatitis or cholecystitis were less commonly referred via this pathway, since cholecystectomy was either performed during the index emergency admission or arranged at discharge. Blood tests including liver function and amylase were routinely performed prior to referral. An information leaflet regarding cholecystectomy was sent to each patient prior to clinic. At the outpatient appointment, each patient was assessed by the surgeon and their suitability for surgery established. Patients with a history of deranged liver function tests and/or bile duct dilatation were investigated preoperatively with magnetic resonance cholangiopancreatography unless contraindicated. One surgeon offered intraoperative laparoscopic ultrasound and bile duct exploration, whilst the remaining surgeons used preoperative endoscopic retrograde cholangiopancreatography (ERCP) for duct clearance as required. Those suitable for surgery were consented, preassessed, and provided with a choice of dates for surgery. Initial day-case criteria were set as follows: Body Mass Index (BMI) less than 35 kg/m^2^, American Society of Anesthesiologists (ASA) grade [2] less than 3, no previous upper abdominal surgery and patient's home within 60 minutes' drive of the hospital. USS findings of a contracted or thick-walled gallbladder were also contraindications to day-case surgery. Following a visit by the NHS Institute for Innovation and Improvement in March 2009 these criteria were relaxed to exclude only patients with deranged liver function tests or who lived greater than 60 minutes drive from the hospital. 

Patients were admitted on the day of surgery. Antibiotics were not used routinely intraoperatively. Patients received either total intravenous or inhalational (volatile) general anaesthesia according to anaesthetists' preference, in addition to intraoperative fentanyl. Postoperative intravenous or intramuscular morphine was avoided, with oramorph used preferentially if required. All patients received two intraoperative antiemetics. Local anaesthesia was injected at the port sites. Dissolvable sutures or glue were preferentially used for skin closure.

Postoperatively patients were managed on the day-surgery recovery ward. Those not suitable for discharge required an inpatient bed, since no twenty-three hour stay facility was available. Patients were discharged with cocodamol 30/500 and a nonsteroidal anti-inflammatory drug (NSAID). Where NSAIDs were contraindicated or patients required greater than two doses of oramorph in recovery, sublingual prochlorperazine and oramorph were used instead, in addition to cocodamol. Routine follow-up outpatient appointment was not offered, however a comprehensive information leaflet was provided to patients on discharge. Where complication arose, patients were advised to call the day-surgery ward between 8 AM and 8 PM or alternatively attend their General Practitioner or Accident and Emergency Department.

Data was collected prospectively for all patients undergoing cholecystectomy, independent of their referral pathway, between 1 January 2007 and 30 June 2009. Patients in whom cholecystectomy was performed as part of a hepatopancreaticobiliary resection were not included in this analysis. The following outcomes were measured: total number of procedures, elective versus emergency, inpatient versus day-case, laparoscopic versus open, conversion rate, and readmission rate within 28 days of surgery. This included an interim audit, which was conducted between 2 September 2008 and 31 October 2008, to examine further the referral source, proposed surgery, timing of surgery, length of stay, and conversion rate. Changes resulting from this audit are presented in the Results section. Additionally a short patient questionnaire was designed to examine postoperative analgesic requirements, incidence of nausea and vomiting, in addition to wound complications. This was administered to 40 consecutive patients postoperatively and their responses returned in a stamp addressed envelope.

The NHS Institute for Innovation and Improvement visited our institution in January and March 2009 to review our patient pathway and facilitate process mapping.

## 3. Results

**Figure 3:**
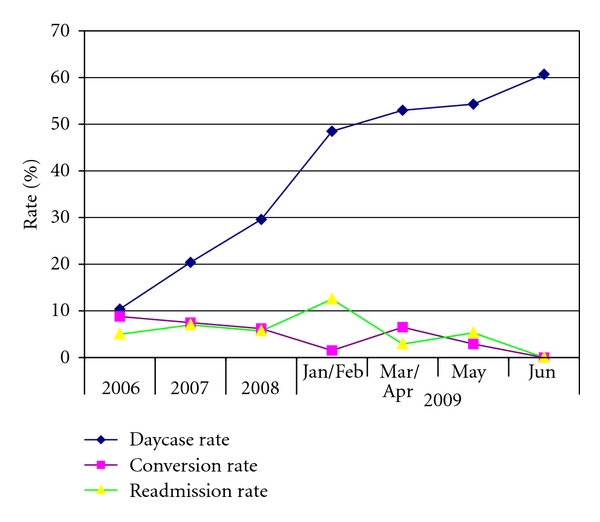
Daycase, conversion and readmission rates following laparoscopic cholecystectomy between January 2006 and June 2009.

A total of 1326 cholecystectomies were performed during the study period (Table 1). 1,130 (85.2 per cent) were performed as an elective and 196 (14.8 per cent) as an emergency procedure. 1,197 (90.2 per cent) were performed laparoscopically and 129 (9.8 per cent) were performed open. 62 (6.1 per cent) elective and 27 (14.5 per cent) emergency laparoscopic procedures were converted to open. 329 (32.5 per cent) elective cholecystectomies were performed as a day case, with an average readmission rate of 4.0 per cent. The number of patients primarily listed for a day-case cholecystectomy increased from 356 in 2006 to 477 in 2008. The laparoscopic rate and day-case rates both increased, with no change in either conversion or readmission rate (Figure 3).

### 3.1. Interim Audit (2 September–31 October 2008)

72 patients underwent cholecystectomy during this period with a mean (range) age of 48 (18–85) years. 19 (26 per cent) were male and 53 (74 per cent) female. 28 (39 per cent) had been listed from a routine surgical outpatient clinic, 27 (38 per cent) from the new specialist-led gallbladder clinic, and 17 (23 per cent) following an emergency admission. 44 (61 per cent) patients were listed as day cases and 28 (39 per cent) for inpatient stay. 6 (9 per cent) patients required conversion to open, of which 3 had previously had an emergency admission. 24 (33 per cent) patients were discharged on the day of surgery. 48 (67 per cent) patients required an inpatient stay, of which 18 would have been suitable for day-case surgery had they not been scheduled on an afternoon operating list. This led to the changes outlined in Table 2.

Following the above changes, the day-case rate increased to 61 per cent in June 2009, with no significant change in laparoscopic, readmission, or conversion rates observed (Figure 3). The number of emergency cholecystectomies performed remained unchanged at around 80 cases per year.

### 3.2. Patient Questionnaire

19 patients returned the patient questionnaire. Overall satisfaction with the service was scored as excellent (*n* = 12), good (*n* = 5), average (*n* = 1), and poor (*n* = 1). The patient scoring “average” had queued outside the ward with other patients on the morning of surgery and the patient scoring “poor” had postoperative pain. No postoperative wound complications were reported.

## 4. Discussion

In the present study the day-case rate following laparoscopic cholecystectomy increased fivefold following the introduction of a new streamlined gallbladder patient pathway, with no associated increase in either conversion rate or readmission rate. Expanding the criteria for day-case surgery and ensuring that patients were scheduled on morning operating lists increased this day-case rate further to 60 per cent.

Day-case laparoscopic cholecystectomy can save costs and has been shown to be a safe and effective treatment for symptomatic gallstones [3–6]. These cost savings primarily arise as a result of reducing unnecessary in-hospital patient stay, which is estimated at *£*249 per day [7]. The day-case rate of 60 per cent achieved in the present study could therefore equate to annual savings of at least *£*74,700 based on a hospital performing 500 cases per year. Higher day-case rates are therefore desirable, although in the context of randomised controlled trials, with patients selected on the basis of operative fitness and proximity to hospital, a day-case rate of only 80 per cent is reported [3, 4, 8–10]. This relates predominantly to uncontrolled pain, nausea, and vomiting, which are known to affect both hospital stay and patient discharge [3, 4]. The use of intraoperative local anaesthetic, postoperative paracetamol, and nonsteroidal anti-inflammatories, with an avoidance of opiates, have all been suggested as techniques to minimise these problems [1]. Since October 2009 our own institution has therefore introduced a standardised anaesthetic and postoperative analgesia protocol for day-case laparoscopic cholecystectomy, which it is hoped will further increase day-case rates. Additional cost savings are also achievable by using an integrated patient pathway, such as that shown in Figure 2, which can minimise the need for repeat ultrasound studies (*£*49), blood tests (*£*10), and outpatient appointments (*£*88) [7]. The use of nondisposable surgical instruments and limiting the use of intraoperative antibiotics is also important. 

The gallbladder pathway used in this study adheres to the principles outlined in the “Focus on Cholecystectomy” document [1]. Reducing the number of patient visits by providing preassessment at the initial clinic visit and preventing routine outpatient followup resulted in less disruption to patients. This is particularly important due to the wide geographical distribution of our patients, although these limitations in access to transport may have also led to some patients not being suitable for day-case surgery. Providing patients with a choice of dates for surgery led to fewer cancellations on the day of surgery. Staggered admission times, whilst preventing long periods of waiting or starvation, were not used during this study. These were limited by the need for an anaesthetist or surgeon to see the patient preoperatively, particularly as operating lists were increasingly pooled to meet waiting list targets. Clerical error, particularly with respect to patients being listed on afternoon operating lists, resulted in a number of patients suitable for day-case surgery requiring an overnight stay. This issue has been previously identified in randomised trials of laparoscopic day-case cholecystectomy versus overnight stay [9]. Following the interim audit in 2008, patients suitable for day-case were predominantly scheduled on a morning list or first on the afternoon list, which resulted in a substantial increase in day-case rates from 30 to over 60 per cent. Increasing the duration of daycase unit opening hours and ensuring patients are discharged according to criteria that do not include set time periods, may enhance this further.

Whilst patient satisfaction and anxiety was not formally assessed in the present study, there is no clear evidence from randomised trials of an increase in anxiety following day-case surgery [5]. Indeed one study found an increased anxiety in those patients randomised to overnight stay [4]. Likewise initial concerns regarding the detection and management of complications in patients discharged on the day of surgery, particularly postoperative bleeding or bile duct injury, have also been unfounded [11]. Major bleeding is uncommon and bile duct injury is predominantly detected at the time of surgery or several days later. The introduction of a telephone follow-up service is therefore proposed at our institution in order to examine patient satisfaction, anxiety, and complication rates as part of a future study.

Readmission rates following day-case cholecystectomy remained relatively unchanged during the study period at around 5 to 7 per cent. This appears higher than the 2 to 3 per cent rate reported in other series [5, 9, 12], however since individual patient data relating to these readmissions was not formally analysed, the reasons for this disparity remain unclear. 

The overall conversion rates in this study of 6.1 and 14.5 per cent following elective and emergency laparoscopic cholecystectomy, respectively, were comparable to those reported nationally [13, 14]. However since 2008 these rates have fallen further to 3.1 and 10.5 per cent, respectively. This is likely to have arisen as a consequence of more cholecystectomies being performed by the five specialist upper gastrointestinal surgeons. Whilst cholecystectomy during index admission with cholecystitis is associated with no significant difference in complication rate or conversion rate [15], it is known to reduce costs, in part due to minimising patient readmission whilst awaiting an elective procedure [1]. Indeed the estimated cost of a patient admitted with acute cholecystitis and treated conservatively is *£*1,875. Despite this, less than 15 per cent of cholecystectomies were performed during an emergency admission in the present study, which is comparable to that reported nationally [14, 16]. Future plans to implement an emergency gallbladder service would facilitate an increase in this proportion.

This study reports the findings of a gallbladder service involving 13 surgeons. There is likely to have been variation in practice due to no clear standardisation of operative technique. Anaesthetic and postoperative analgesia regimes may have varied according to anaesthetist preference and a standardised gallbladder anaesthetic pathway was not introduced until after completion of this study. Postoperative complications rates are not reported here since these were not directly measured. Less than 50% of patients returned the patient questionnaire and therefore results must be interpreted with caution. Likewise patient satisfaction and anxiety were not directly measured, however this is part of an ongoing study.

## 5. Conclusion

Implementing a standardised patient pathway for day-case laparoscopic cholecystectomy has increased day-case rates sixfold, with no associated increase in readmission or conversion rate. Engagement with clerical, nursing, and medical staff, in addition to management of patients' expectations following surgery was a vital part of this process. Future standardisation of anaesthetic and analgesic regimes may improve this further.

##  Conflict of Interests

The authors have no conflict of interests to declare.

## Figures and Tables

**Table 1:** Surgical factors relating to cholecystectomies performed between 2006 and 2008.

		2006	2007	2008	Jan/Feb	Mar/Apr	May	June
					2009	2009	2009	2009
TOTAL		464	512	578	87	78	37	34
Elective	Open	15	12	12	2	3	0	1
	Laparoscopic	327	373	449	65	62	34	28
Emergency		79	86	81	17	6	1	5

**Table 2:** Changes made to gallbladder pathway following interim audit in 2008.

(i) All day-case cholecystectomies on morning list or first on afternoon list	
(ii) Default to day-case unless social reasons, deranged liver function or anaesthetist/surgeon choice	
(iii) Remove BMI and previous upper abdominal surgery as day-case criteria	
(iv) Increase patient referral through “gallbladder clinic”	
